# A specific allele of *MYB14* in grapevine correlates with high stilbene inducibility triggered by Al^3+^ and UV-C radiation

**DOI:** 10.1007/s00299-018-2347-9

**Published:** 2018-10-09

**Authors:** Ru Bai, Yangyang Luo, Lixin Wang, Jing Li, Kerun Wu, Guifang Zhao, Dong Duan

**Affiliations:** 1 Key Laboratory of Resource Biology and Biotechnology in Western China, Ministry of Education, College of Life Sciences, Northwest University, Xi’an, 710069 China; 2 Research Center of Chinese Jujube, Agricultural University of Hebei, Baoding, 071001 Hebei China

**Keywords:** Defence, Grapevines, *MYB14*, Stilbene accumulation, Al^3+^, UV-C

## Abstract

**Key message:**

The structural differences of *MYB14* promoter in two grapevine genotypes affect the expression of *MYB14* and stilbene synthesis in response to Al^3+^ and UV-C radiation.

**Abstract:**

Grapevines provide an important fruit crop worldwide, but production is often limited by pathogen infection. Stilbenes, a class of secondary metabolite, represent phytoalexins that contribute to defence against pathogens in many plants, including grapevine. It is known that the transcription factors *MYB14* and *MYB15* are required for the activation of the promoters of resveratrol synthase to regulate stilbene biosynthesis. In the current study, we observed that stilbene levels were more highly induced by Al^3+^ and UV-C radiation treatments in the cultivar *Vitis labrusca* ‘Concord’ than in the cultivar *V. vinifera* ‘Cabernet Sauvignon’. We investigated whether genetic/structural variations in the *MYB14* and *MYB15* promoters between these two representative genotypes are responsible for the differences in stilbene accumulation. Significant differences in the structure and activity of the promoter of *MYB14*, but not *MYB15* were identified between the two genotypes, following heterologous expression in *Nicotiana benthamiana* system and treatments with Al^3+^ and UV-C. Hydrogen peroxide (H_2_O_2_) was detected in Concord soon after the stress treatments, but after diphenyleneiodonium chloride pre-treatment, the expressing level of *VlMYB14*, the promoter activity of *VlMYB14* and the accumulation of stilbenes was significantly reduced. A model is presented where the induction of *MYB14* contributes to stilbene accumulation in Concord following Al^3+^ and UV-C treatments involving reactive oxygen species (ROS) production as an early signal.

**Electronic supplementary material:**

The online version of this article (10.1007/s00299-018-2347-9) contains supplementary material, which is available to authorized users.

## Introduction

Grapevine (*Vitis* spp.) provides an economically important fruit crop and cultivars are grown in temperate areas worldwide. However, production can be severely restricted by environmental factors and so a range of distinctive defence responses to various biotic and abiotic stresses, especially to pathogen attack, has been studied in order to enhance yield. American grapevines are generally more resistant to pathogens than are the genotypes from Europe. For instance, *V. labrusca* ‘Concord’ is characterized as a pathogen-resistant genotype (Pearson and Gadoury 1992; Pearson and Goheen 1988), while *V. vinifera* ‘Cabernet Sauvignon’ is relatively pathogen-sensitive (Boso and Kassemeyer 2008; Marsh et al. 2010). However, due to the extended coevolution of grapevines and pathogens, some American grapevines, such as the *V. labrusca* ‘Concord’, which is known for its ability to resist the destructive disease ‘powdery mildew’, now suffer from infection by pathogens, such as *Plasmopara viticola* strains (Gadoury et al. 2001; Gómez-Zeledón et al. 2013; Rouxel et al. 2013). There is thus a growing need to characterize and deploy mechanisms of resistance to pathogens in grapevines.

Stilbenes are important secondary metabolites in plants, including grapevine, where they act as phytoalexins and improve immunity to disease (Langcake and Pryce 1976; Kodan et al. 2001; Yu et al. 2005). However, stilbenes have recently attracted attention not only for their defensive roles in plants, but also for their pharmacological value and beneficial effects on human health (Vannozzi et al. 2012). Stilbenes synthesis, via the phenylpropanoid pathway (Höll et al. 2013), can be induced in response to biotic and abiotic stresses, such as pathogen attack (Schnee et al. 2008; Adrian et al. 1996, 1997), UV-C radiation (Bais et al. 2000) and application of chemicals, including aluminium ions (Adrian et al. 1996). However, even though a number of transcription factors have been demonstrated to play key roles in the accumulation of stilbenes, the underlying molecular mechanisms are still not well characterized.

Wong and Matus (2017) reviewed the integrated molecular network of phenylpropanoid regulation in the grape berry, including the relationship between enzyme coding genes and several transcription factor (TF) families (MYB, bZIP, WRKY, AP2/ERF and bHLH), long non-coding RNAs (lncRNAs), and micro RNAs (miRNAs). With regard to stilbene accumulation, R2R3-MYB-type TFs have been widely studied (Höll et al. 2013) and two of them, *MYB14* and *MYB15* from grapevine, have been shown to activate the promoters of stilbene synthase and be involved in stilbene biosynthesis. *MYB13*, an uncharacterized close homologue of *MYB15*, may also be involved in the accumulation of stilbene in different organs and in response to biotic and abiotic stresses through co-operation with other TF families (Wong et al. 2016). Recently, WRKY transcription factors were also reported to regulate stilbene synthesis pathway: *VviWRKY24* was shown to enhance the promoter activity of *VviSTS29* to regulate the stilbene synthesis (Vannozzi et al. 2018). Stilbene synthesis is thus controlled by a complex regulatory system that likely involves multiple TFs and regulatory conditions that have yet to be characterized. Here, we investigated the regulation of stilbene biosynthesis by MYB TFs, based on the results of previous studies (Duan et al. 2015, 2016). We identified differences in the inducibility of the *MYB14* promoter that could account for the stress-specific differences in the expression of stilbene synthase among *V. vinifera* ssp. Sylvestris, which is the ancestor of cultivated grapevine in European. We speculate that structural variations between the *MYB14* and *MYB15* promoters may contribute to the differences in stilbene inducibility, as well in other grapevines, including genotypes from North America and cultivars in Europe.

Reactive oxygen species (ROS) are produced as a result of a perturbed redox balance in response to various biotic and abiotic stresses (Apel and Hirt 2004; Boscolo et al. 2003; Wojtaszek 1997; Yamamoto et al. 2003; Ghanati et al. 2005; Gao et al. 2008; Ke et al. 2010). Recent research has indicated that ROS not only function as toxic compounds that damage cells (Buchanan et al. 2000), but also act important early signal molecules (Fath et al. 2002; Vranová et al. 2002; Wang and Nick 2017). Importantly, ROS have been proposed to be involved in the production of phytoalexins and are necessary for the induction of stilbene synthase (Rustérucci et al. 1996; Mithöfer et al. 1997; Chang et al. 2011; Duan et al. 2016).

In this current study, we choose two representative genotypes, *V. labrusca* ‘Concord’, which is native to North America and widely planted in the United States, and *V. vinifera* ‘Cabernet Sauvignon’, which is a common cultivated in Europe. Both cultivars are well known sources of grape juice and wine. We used two stress treatments, UV-C radiation and Al^3+^, which are potent elicitors (Adrian et al. 1996; Ahad and Nick 2007; Duan et al. 2015), to induce the stilbene synthesis in grapevines tested the hypothesis that genetic diversity between the *MYB14* and *MYB15* promoters is responsible for the differential accumulation of stilbenes between the grapevine genotypes. The overarching longer-term objective is to develop methods to increase resistance of grapevines to fungal pathogens through environmentally benign approaches, avoiding the use of fungicides. Our results reveal a specific allele of *MYB14* in Concord that is a potential target for improving resistance to pathogens.

## Materials and methods

### Plant materials

Two grapevine genotypes, *V. labrusca* ‘Concord’ and *V. vinifera* ‘Cabernet Sauvignon’, were cultivated in the Life Science Experimental Park of Northwest University, Xi’an, Shaanxi, China. *Nicotiana benthamiana* plants were grown in growth cabinets with controlled 16 h light/8 h dark period at 23 °C. The third to fifth fully-expanded leaves from the plant apex of Concord and Cabernet Sauvignon were randomly selected for treatment with UV-C and aluminium chloride. For the UV-C treatment, leaves were placed upside down on moist filter paper in petri dishes and the abaxial surface of an entire leaf was exposed to UV-C light (254 nm, 15 W, FSL, China) for 10 min at a distance of 12.5 cm from the light source. For treatment with aluminium chloride, leaves were separately placed in petri dishes on filter paper and soaked in 15 mL freshly prepared 1.0% AlCl_3_ solution (Tianli Chemical Reagent Co., Ltd, China). The same experimental treatment, but with sterile water rather than AlCl_3,_ was performed as a negative control. After that, the leaves of Concord and Cabernet Sauvignon were harvested at different time points, immediately frozen with liquid nitrogen and then stored at − 80 °C for RNA extraction or stilbene, H_2_O_2_ and malondialdehyde (MDA) analysis.

### Stilbene analysis and quantification

For the UV-C treatment, leaves of Concord and Cabernet Sauvignon were collected at the following time points: C (control fresh leaf, without UV-C treatment), 6 h and 24 h (from the end of the 10 min UV-C pulse), respectively. For the aluminium chloride treatment, leaves of two genotypes were collected at the following time points: C (control fresh leaf, without aluminium chloride treatment), 6, 12, 24, and 48 h. All of the sterile water treated samples were collected as a negative control at the corresponding time points. Stilbenes were extracted as described by Duan et al. (2015) and analysed using high-performance liquid chromatography (HPLC, Waters 2696, America). Chromatographic separations were performed on an Agilent ZORBAX SB-C18 (5 µm, 4.6 × 250 mm) column maintained at room temperature. The mobile phase consisted of acetonitrile (eluent A) and phosphoric acid (0.1%, v/v) (eluent B) at a flow rate of 0.8 mL min^−1^. The gradient elution program was as follows: 0–20 min, 15–30% A; 20–35 min, 30–50% A; 35–45 min, 50% A. The injecting volume was 20 µL. The peak areas were recorded and calculated busing an external standard method.

### Determination of H_2_O_2_ and MDA in grapevine leaves

The leaves of Concord and Cabernet Sauvignon were harvested at different time points after the UV-C irradiation: C (control fresh leaf, without UV-C treatment), 0, 10, 15, 30 and 60 min after the 10 min UV-C pulse. The time points for the aluminium chloride treatment as follows: C (control fresh leaf, without 1.0% AlCl_3_ solution), 15, 30, 60, 90 and 120 min. All of the sterile water treated samples were used as a negative control at the corresponding time points. Levels of H_2_O_2_ in leaves were determined as described by Wang et al. (2017), by using a peroxide assay kit (Comin Biotechnology Co., Ltd. Suzhou, China). Hydrogen peroxide (H_2_O_2_) is one of the most important ROS. Lipid peroxidation as readout for oxidative burst was determined by measuring the production of MDA as previously described (Hodgson and Raison 1991), but with minor changes: the plant leaves (100 mg) were ground in liquid nitrogen using a pestle and mortar, vortexed for 45 s in 1 mL 0.1 M phosphate buffer (pH 7.4) in a 2.0 mL Eppendorf (EP) tube, centrifuged for 4 min at 8000*g* and the pellet discarded. The remaining 200 µL of supernatant were added to a reaction mixture containing 750 µL acetic acid (20% w/v), 750 µL thiobarbituric acid (TBA) (aqueous solution, 0.8% w/v), 200 µL Milli-Q water and 100 µL sodium dodecyl sulphate (8.1% w/v). An identical reaction mixture, where the supernatant from the sample was replaced by an equal volume of phosphate buffer, was used as a control. The reaction mixture was then incubated for 1 h at 98 °C and cooled to room temperature. The absorbance of the solution at 535 nm (specific signal) and 600 nm (background) were measure using a microplate reader (Synergy 2, BioTek, America). Lipid peroxidation was calculated as µM MDA from A535 to A600 using an extinction coefficient of 155 mM^−1^ cm^−1^.

To further investigate potential ROS signalling, leaves of Concord were treated with 10 min UV-C, 1% AlCl_3_, or 1% H_2_O_2_ (w/v) (FuYu Fine Chemical Co., Ltd., China). The leaves were harvested at 24 h post-treatment. For the diphenyleneiodonium chloride (DPI) (Sigma-Aldrich, shanghai) treatment, the leaves were pretreated with 100 µM DPI by soaking for 1 h before the UV-C, Al^3+^ or H_2_O_2_ treatments. DPI was also added without a subsequent treatment to evaluate the effect of the inhibitor alone. Leaves treated with sterile water in which the AlCl_3_ and H_2_O_2_ were dissolved served as negative controls. After the treatments, the leaves were immediately frozen in liquid nitrogen and stored at − 80 °C for RNA extraction and stilbene analysis. Three samples were treated in each treatment, and each treatment was repeated three times.

### Isolation of *MYB14*/*15* promoter fragments and sequence analysis

Genomic DNA was extracted from the leaves of both grapevine genotypes using a new rapid plant genomic DNA extraction kit (BioTeke, Beijing, China) according to the manufacturer’s protocol. MYB14-F: (5′-CTACTGACGTGCACTAGCCT-3′), MYB14-R: (5′-GCAGAGTGAAAGTGCAACACG-3′) and MYB15-F: (5′-GCCAAGGACTTGACTTGGAA-3′) and MYB15-R: (5′-CTTCGATGACCAAATCTTTGAA-3′). PCR primers were used to amplify the full length *MYB14* and *MYB15* genes respectively, using genomic DNA as a template. Fragments were amplified using LA Taq DNA Polymerase (TaKaRa) following the manufacturer’s recommended reaction conditions. The PCR products were cloned into T-Vector pMD™ 19 (Simple) (TaKaRa, Dalian, China) and the resulting constructs were transformed into *Escherichia coli* DH5α competent cells to produce pMD-*VvMYB14*/*15* and pMD-*VlMYB14*/*15*, which were then sequenced (Sangon Biotech, Shanghai, China). PCR primers were designed to amplify the promoters with restriction enzyme sites *Hin*dIII and *Bgl*II (Table S1). The PCR products were respectively linked to T-Vector pMD™19 to produce pMD-*pVvMYB14*/*15* and pMD-*pVlMYB14*/*15*, which were then sequenced. Potential *cis*-elements in the promoter sequences were predicted with the online software packages Plant CARE (http://bioinformatics.psb.ugent.be/webtools/plantcare/html/) and PlantPan 2.0 (http://plantpan2.itps.ncku.edu.tw/promoter.php).

### *Agrobacterium*-mediated transient assay

Four expression vectors, *pVvMYB14*::GUS, *pVlMYB14*::GUS, *pVvMYB15*::GUS and *pVlMYB15*::GUS, were constructed for transient expression assays. The promoter sequences *pVvMYB14*/*15* and *pVlMYB14*/*15* were cloned into pCAMBIA1301 expression vector upstream of the β-glucuronidase (GUS) reporter (http://www.miaolingbio.com/plasmid/P0277.html). Each construct was individually introduced into *Agrobacterium tumefaciens* strain GV3101 by electroporation (Mersereau et al. 1990). The resulting *Agrobacterium* stains were cultivated in the dark on a shaker (ZHCHENG, Shanghai) at 200 rpm for 24 h at 28 °C then centrifuged at 2773*g* for 10 min at room temperature. The pellets were re-suspended in 10 mL of permeation buffer (250 mg d-glucose, 5 mL 500 mM MES, 5 mL 20 mM Na_3_PO_4_·12H_2_O, 5 µL 1 M acetosyringone adjusted to a final volume of 50 mL with ddH_2_O). The bacterial suspensions were incubated at 28 °C for 3 h and the absorbance at OD600, measured using a Nicolet Evolution 754 UV–Vis spectrophotometer (JINGHUA, Shanghai), and adjusted to 0.6 with permeation buffer.

The bacterial suspensions were then injected into *N. benthamiana* leaves that had grown for 6–8 weeks with a needle-free syringe. After 48 h, the leaves were subjected to the various treatments as described as above. For the UV-C treatment, leaves were collected at C and 24 h with the negative control at the corresponding time points. For the aluminium chloride treatment, leaves were collected at C, 6, 12 and 24 h with sterile water treatment used as a parallel negative control. For the DPI pre-treatment experiments, the *N. benthamiana* leaves were pre-treated with 100 µM DPI for 30 min as described in Duan et al. (2016), followed by treatments with Al^3+^ and UV-C for 60 min and 30 min respectively. X-Gluc staining, GUS expression and GUS enzymatic activity were analysed as described by Xu et al. (2010) and Jiao et al. (2016).

### cDNA synthesis and quantitative real-time PCR

Total RNA from both grapevine genotypes and *N. benthamiana* leaves was extracted using an EZNA® Total RNA kit (Omega Bio-tech) according to the manufacturer’s instructions. mRNA was transcribed into cDNA using Prime Script Reverse Transcriptase (TaKaRa). Quantitative real-time PCR was conducted as described in Xu et al. (2011). *NbEF1-α* (for *N. benthamiana* leaves) (Zhang et al. 2015) and *EF1-α* (for grapevine leaves) (Duan et al. 2015) were used for gene expression normalization and marker genes were used for comparison (Belhadj et al. 2008; Xu et al. 2010; Höll et al. 2013; Duan et al. 2015, 2016). Primers used in the experiment are shown in Table S2. Each experiment was carried out in three biological repetitions.

## Results

### Stilbenes are highly induced in Concord compared to Cabernet Sauvignon following UV-C or Al^3+^ treatments

**Fig. 1:**
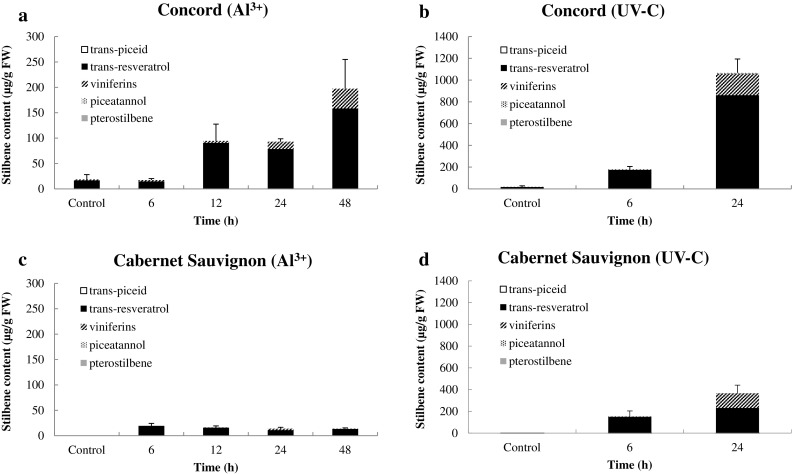
Time courses of stilbene accumulation in *V. labrusca* cv. Concord and *V. vinifera* cv. Cabernet Sauvignon in response to UV-C or aluminium chloride. Leaves of Concord and Cabernet Sauvignon were harvested at various time points after treatment with 1.0% aluminium chloride (**a, c**) or 10 min exposure to UV-C (**b, d**). Data represent mean values and standard errors from three independent biological replicates

Stilbene biosynthesis is known to be induced by different stresses, including UV-C (Duan et al. 2015) and AlCl_3_ (Adrian et al. 1996), and in this study we examined their effects on stilbene accumulation in Concord and Cabernet Sauvignon. As shown in Fig. 1, following either treatment *trans*-resveratrol and viniferins were the main stilbenes in leaves and we noted especially high levels of *trans*-resveratrol. Stilbene accumulation was rapidly induced to higher levels in Concord than in Cabernet Sauvignon. For example, 24 h following UV-C treatment, we detected 860 µg g^−1^ fresh weight (FW) of *trans*-resveratrol and 200 µg g^−1^ FW of viniferins in Concord, compared with 230 µg g^−1^ FW and 135 µg g^−1^ FW, respectively, in Cabernet Sauvignon (Fig. 1b, d). Similarly, an increase in stilbene levels was observed following AlCl_3_ treatment from 6 to 48 h in Concord (Fig. 1a), whereas no obvious changes were observed over time in Cabernet Sauvignon (Fig. 1c). UV-C treatment induced greater stilbene accumulation than did the Al^3+^ treatment at same time points in both genotypes. For example, total stilbenes levels reached 1200 µg g^−1^ FW 24 h after UV-C treatment in Concord, (Fig. 1b), while the Al^3+^ treatment resulted in 100 µg g^−1^ FW (Fig. 1a). The accumulation of *trans*-resveratrol was detected at early time points (e.g. 6 h) in both genotypes and treatments, while the viniferins accumulated later and were most abundant at 24 h.

### The expression levels of key stilbene-related genes are higher in Concord than in Cabernet Sauvignon following Al^3+^ or UV-C treatments

**Fig. 2:**
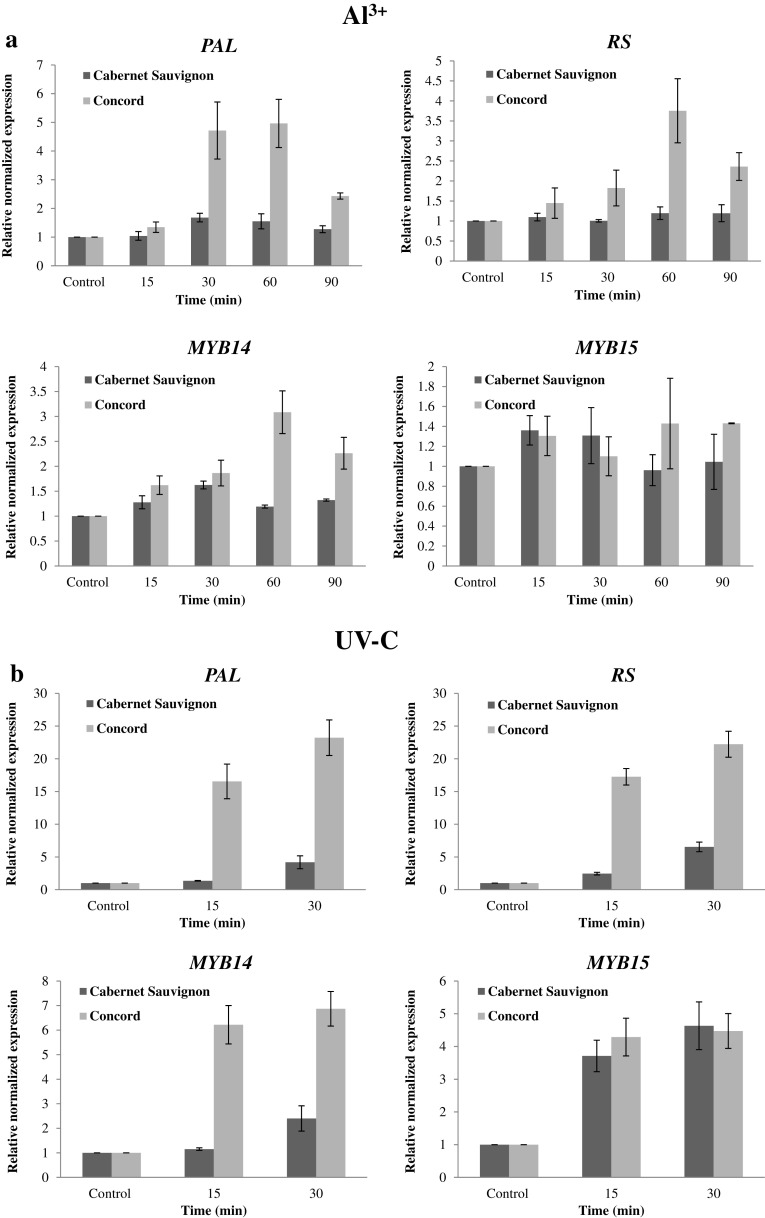
Expression analysis of phenylalanine ammonium lyase (*PAL*), resveratrol synthase (*RS*), *MYB14* and *MYB15* in response to Al^3+^ (**a**) or UV-C (**b**) treatments in leaves of Cabernet Sauvignon and Concord by real-time quantitative PCR. Data represent mean values from three independent experimental series and error bars represent standard errors

We investigated whether the different accumulation of stilbenes correlated with the expression of key genes involved in stilbene synthesis pathway, including the upstream enzyme in the phenylpropanoid pathway phenylalanine ammonium lyase (*PAL*), *trans*-resveratrol synthase (*RS*), as well as *MYB14* and *MYB 15*, which are known to regulate the expression of resveratrol synthase (Höll et al. 2013; Duan et al. 2016). The transcript levels of *PAL, RS, MYB14* and *MYB15* were investigated by real-time quantitative PCR (RT-qPCR) at the early time points in leaves of Cabernet Sauvignon and Concord following Al^3+^ and UV-C treatments. Following the Al^3+^ treatment, the expression of *PAL* and *RS* in Concord increased from 30 min (five- and twofold, respectively) and peaked at 60 min (Fig. 2a). The expression of *MYB14* showed the same pattern and had maximal levels at 60 min (almost threefold compared with control). However, these responses were less pronounced in Cabernet Sauvignon. Transcript levels of *PAL, RS* and *MYB14* were rapidly induced after the 10 min UV-C pulse in Concord (Fig. 2b), whereas the induction of these transcripts in Cabernet Sauvignon was relatively slower and peaked at lower levels. Regardless of the expression levels, the induction levels of *MYB15* showed no significant difference between Cabernet Sauvignon and Concord for both treatments.

### Differences in the *MYB14* promoter sequences between Concord and Cabernet Sauvignon

**Fig. 3:**
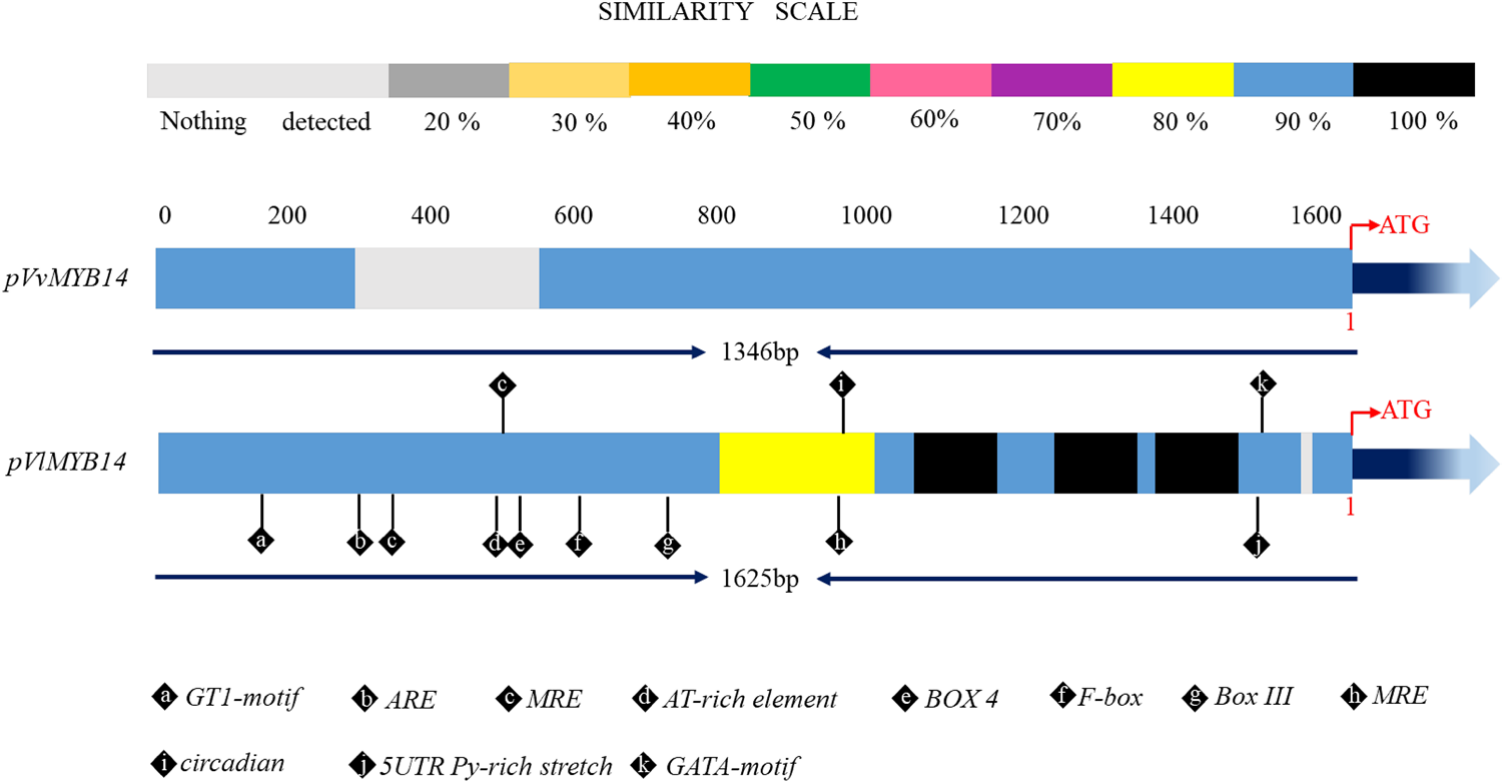
Differences in the *MYB14* promoter sequences between Concord and Cabernet Sauvignon, and predicted *cis*-acting elements

We next investigated whether differences in the *MYB14* and *MYB15* promoter sequences from the two genotypes might be responsible for the differential induction of *RS*, thereby influencing stilbene diversity. As indicated in Fig. 3, we identified a large deletion (300 bp) in *pVvMYB14* compared to *pVlMYB14*; however, the sequences shared 99% nucleic acid identity and showed no other differences between *pVvMYB15* and *pVlMYB15* (Fig. S1).

To identify possible regulatory *cis*-acting elements in the promoters, both *pVvMYB14* and *pVlMYB14* were analysed using the PlantCARE algorithm (Lescot et al. 2002). As shown in Fig. 3 and Fig. S2, the promoter region of *VlMYB14* was predicted to have more *cis*-elements than *VvMYB14*. For example, the 5′-UTR Py-rich stretch has been reported to confer high transcriptional levels (Daraselia et al. 1996; Wang et al. 2013); the TATA-box is a sequence of DNA found in the core promoter region of genes (Bae et al. 2015; Whittington et al. 2008); the AT-rich element acts as an enhancer (Bustos et al. 1989; Sandhu et al. 1998); Box III is predicted to be a protein binding site involved in salt stress responses (Sun et al. 2010) and several *cis*-elements (e.g. a GATA-motif, GT1-motif and MRE) are linked to light responsiveness. In addition, the transcription factor binding sites (TFBSs) in the promoter sequences of *pVvMYB14* and *pVlMYB14* were analysed using PlantPAN 2.0 (Chow et al. 2016). The transcription factors that were specific to *Vitis* were selected. In general, more TFBSs were identified in *pVlMYB14* than in *pVvMYB14* (Table S3). Several sequence motifs that are present in the binding sites of some key transcription factors, such as WRKY, bHLH, bZIP, NAC, AP2-ERF, Alpha-amylase, AT-Hook, C2H2, Dof, GATA, MADF/Trihelix, and that might play roles in growth and development, hormone regulation and stress response were identified.

### The promoter induction is stronger in *VlMYB14* than in *VvMYB14* induced by Al^3+^ and UV-C

**Fig. 4:**
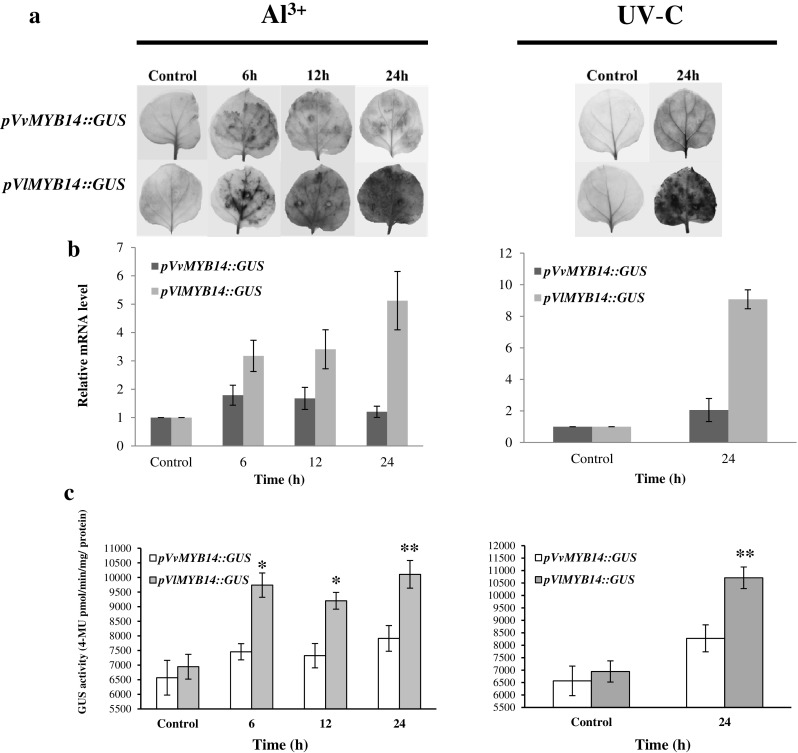
Time course of the heterologous expression of the GUS reporter following introduction of *pVvMYB14*::GUS or *pVlMYB14*::GUS in transgenic *N. benthamiana* leaves in response to Al^3+^ and UV-C treatments. **a** Histochemical assay of GUS expression in the transiently transformed *N. benthamiana* leaves. **b** GUS transcript abundance in response to 1% Al^3+^ treatment at different time points and at 24 h after UV-C irradiation for 10 min, measured by RT-qPCR. **c** GUS enzymatic activity. Values represent mean values and standard errors from three independent experimental series. *P < 0.05 and **P < 0.01 indicates statistical significant differences between Cabernet Sauvignon and Concord at the same time points (*n* = 3)

To investigate the functional significance of the structural differences between *pVlMYB14* and *pVvMYB14*, the 1625 bp (*VlMYB14*) and 1346 bp (*VvMYB14*) promoter fragments were inserted into the vector pCAMBIA1301, replacing the *Cauliflower mosaic virus* (*CaMV*) *35S* promoter region, upstream of a GUS reporter (Xu et al. 2010), and transiently expressed in *N. benthamiana*. GUS expression was monitored by X-Gluc staining (Fig. 4a), the RT-qPCR analysis of GUS transcript levels (Fig. 4b) and the quantification of GUS enzymatic activity (Fig. 4c). As shown in Fig. 4a, *pVlMYB14* was more strongly induced than *pVvMYB14* following Al^3+^ or UV-C treatments. Quantification of GUS transcript abundance (Fig. 4b) and enzymatic activity (Fig. 4c) in *pVlMYB14* revealed a strong induction at 6 h after incubation with Al^3+^, which continuously increased until 24 h. In contrast, the induction of *pVvMYB14*::GUS increased slightly but did not show a statistically significant difference during the same time period. Similar response patterns were observed for *pVlMYB14* and *pVvMYB14* when exposed to UV-C irradiation (Fig. 4b and c). *pVlMYB14* was highly induced in the 24 h treatment (~ ninefold), while the induction of *pVvMYB14* remained below the significance threshold. In addition, RT-qPCR analysis of GUS transcript levels driven by *pVlMYB15* and *pVvMYB15* indicated that neither of the *MYB15* promoters was activated by Al^3+^ or UV-C (Fig. S3). These results suggest that the promoter induction following Al^3+^ and UV-C treatments is correlated with the promoter structural differences in the heterologous *N. benthamiana* system, and that the specific allele of *pVlMYB14* may contribute to the high stilbene inducibility.

### ROS levels are induced in Concord by Al^3+^ or UV-C

ROS are known to act as signals that orchestrate cellular adaptations to stresses. To investigate whether ROS might contribute to the activation of *VlMYB14*, we examined the effects of the Al^3+^ and UV-C treatments on ROS production. Specifically, we measured the production of hydrogen peroxide (H_2_O_2_), one of the major ROS species, and the production of MDA, one of the main products of stress-induced membrane lipid peroxidation (Draper and Hadley 1990; Janero 1990).

**Fig. 5:**
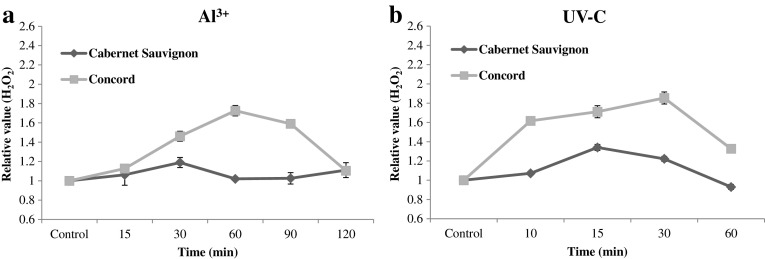
Hydrogen peroxide (H_2_O_2_) levels in Cabernet Sauvignon and Concord leaves following 1% Al^3+^ (**a**) and 10 min UV-C (**b**) treatments. As negative controls, leaves were incubated with sterile water, or not exposed to UV-C. Values represent means and standard errors from nine independent biological replicates

As shown in Fig. 5 and S4, both the Al^3+^ and UV-C treatments induced a rapid production of H_2_O_2_ and MDA in Concord, but not in Cabernet Sauvignon. Following the Al^3+^ treatment (Fig. 5a and Fig. S4a), levels of H_2_O_2_/MDA increased from around 15 min and peaked at 60 min in Concord, then sharply declined from 60 to 120 min and approached control levels at 120 min. A similar pattern was observed after UV-C irradiation in Concord, but the maximal levels of H_2_O_2_ and MDA were significantly higher and the induction was more rapid than following the Al^3+^ treatment (Fig. 5b and Fig. S4b).

### ROS are necessary for the activation of *pVlMYB14*, induction of *VlMYB14* and accumulation of stilbenes

**Fig. 6:**
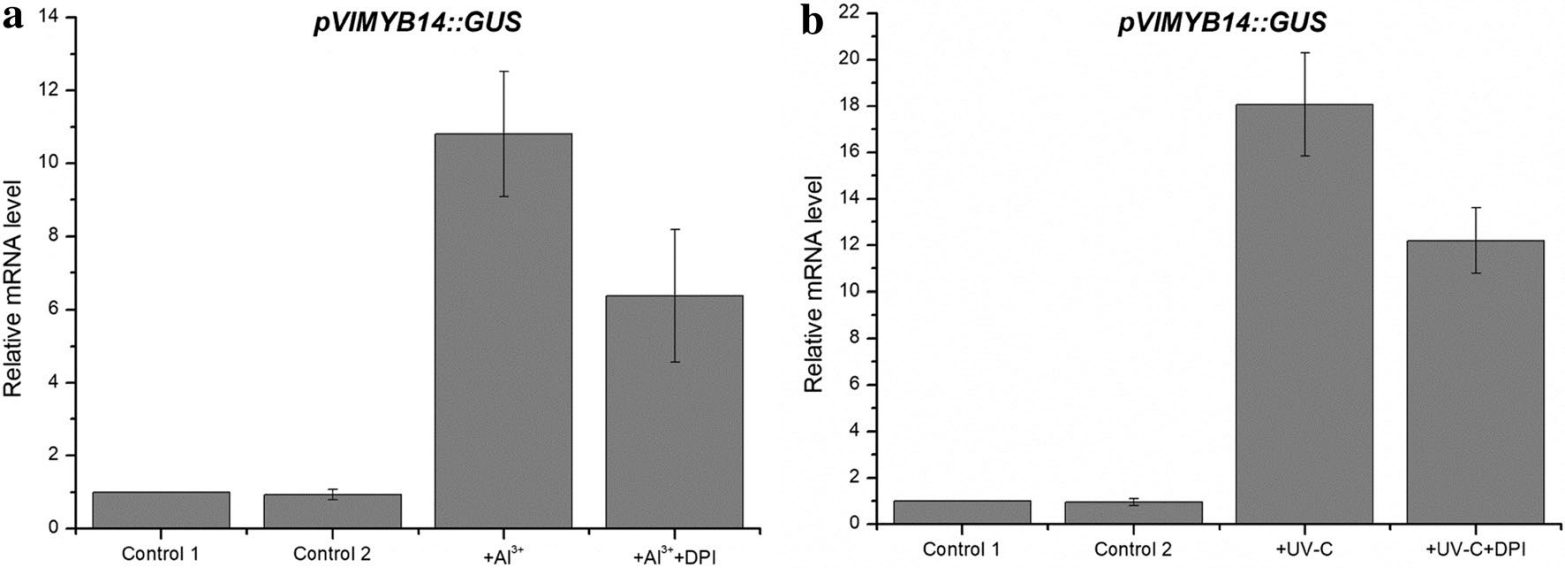
GUS transcript levels, resulting from the expression of *pVlMYB14*::GUS in response to Al^3+^ and UV-C treatments in combination with DPI pretreatment, measured by RT-qPCR. **a** Mean value of the fold induction levels of the promoter in response to 1% aluminium chloride without/with DPI pretreatment. Control 1 and 2 were pretreated with DMSO (− DPI)/(+ DPI) for 30 min and then kept for 1 h, respectively. Al^3+^ and Al^3+^ + DPI: the relative promoter activity in the presence of 1% Al^3+^ for 1 h without/with pretreatment of DPI (100 µM) for 30 min. **b** Values indicate fold induction of promoter activity in the presence of UV-C and UV-C with DPI pretreatment. Control 1 and 2 were treated with DMSO (− DPI)/(+ DPI) for 30 min, respectively. UV-C and UV-C + DPI: fold induction of promoter activity for 0.5 h without/with DPI (100 µM) after 10 min UV-C irradiation. Values indicate mean values and standard errors from three independent experimental series

To investigate whether the induction of ROS triggered by the Al^3+^ or UV-C are necessary for the activation of *pVlMYB14* in Concord, we measured the activity of *pVlMYB14* in the heterologous *N. benthamiana* system following treatment with DPI, a specific inhibitor of NADPH oxidase. As shown in Fig. 6, we observed that *pVlMYB14* was strongly induced after treatment with Al^3+^ or UV-C. However, after a DPI pretreatment, the induction of *pVlMYB14* was substantially decreased, whereas treatment with DPI alone did not affect the modulation of *pVlMYB14* expression.

**Fig. 7:**
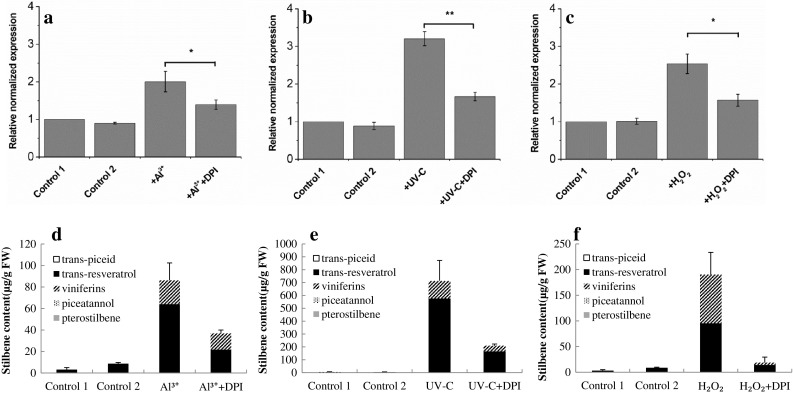
Regulation of *VlMYB14* and stilbene biosynthesis in Concord by Al^3+^, UV-C and H_2_O_2_ in combination with DPI treatment. **a**–**c** Induction of *VlMYB14* in response to Al^3+^/UV-C/1% H_2_O_2_ (w/v) without/with DPI pretreatment. Control 1 and 2 were pretreated with DMSO (− DPI)/(+ DPI) for 1 h and then kept for 24 h. Al^3+^/UV-C/H_2_O_2_ and Al^3+^/UV-C/H_2_O_2_ + DPI: *VlMYB14* transcript levels measured after treatment with Al^3+^ or UV-C/H_2_O_2_ for 24 h without/with pretreatment of DPI (100 µM) for 1 h. **d**–**f** Stilbene levels in Concord at 24 h. The leaves were treated as above. Data represent mean values and standard errors from three independent biological replicates. *P < 0.05 and **P < 0.01 indicate statistically significant differences

In addition to Al^3+^/UV-C, we introduced exogenous H_2_O_2_ as a ROS-donor to investigate the effects on *MYB14* expression. As shown in Fig. 7a–c, compared to the solvent control, the accumulation of *VlMYB14* transcripts significantly increased after the treatments with Al^3+^/UV-C/H_2_O_2_. However, when the leaves of Concord were pre-treated with DPI for 1 h before the Al^3+^/UV-C/H_2_O_2_ treatment, *VlMYB14* transcript levels were higher than those in the solvent control, but significantly lower than in groups that were treated with Al^3+^/UV-C/H_2_O_2_ alone. DPI itself did not affect the expression of *VlMYB14*. These results indicate that *VlMYB14* acts downstream of ROS signalling in Concord.

We also measured stilbene accumulation following with DPI pretreatment prior to the Al^3+^/UV-C/H_2_O_2_ treatments. We found that stilbene accumulation increased significantly after the treatments with Al^3+^/UV-C/H_2_O_2_, but that this effect was strongly suppressed by DPI (Fig. 7d–f). Taken together, the data indicate that ROS enhance the activation of *pVlMYB14* by the Al^3+^ or UV-C treatments and are necessary for stilbene synthesis in Concord.

## Discussion

In the current study, we examined the functional significance of the *MYB14* and *MYB15* in the context of stilbene accumulation in two grapevine genotypes. We observed that the activation of *pVlMBY14*, but not *pVvMYB14*, was strongly induced by Al^3+^ and UV-C treatments, and that these inductions were associated with ROS accumulation. We detected no differences in the sequences or activities of the *MBY15* promoters; however, we identified genetic variations in the *MYB14* promoters between Concord and Cabernet Sauvignon that correlated with stilbene levels. Higher stilbene levels were present in Concord associated with a specific *MYB14* promoter structure and corresponding promoter induction.

Most stilbenes are derivatives of the basic unit *trans*-resveratrol (3,5,4′-trihydroxy-*trans*-stilbene), with variants resulting from differences in the glycosylation pattern, *trans*-piceid, oxidation pattern viniferins and the methylated form pterostilbene. In general, resveratrol and viniferins are the main active and antimicrobial stilbenes (Jeandet et al. 2002). In this study, we observed that *trans*-resveratrol and viniferins represent the primary stilbenes associated with both treatments in the two genotypes. In addition, UV-C triggered more rapid accumulation and higher overall levels of stilbenes than did the Al^3+^ treatment. Additionally, the pattern of stilbene accumulation was time dependent: *trans*-resveratrol accumulated earlier, whereas the viniferins were found later, which is consistent with our previous studies of *V. sylvestris* (Duan et al. 2015).

*MYB14* and *MYB15* are known to activate stilbene/resveratrol synthase and to contribute to stilbene accumulation (Höll et al. 2013); however, other factors are likely involved, such as members of the WRKY TF family. A close functional association between MYB and WRKY TFs has been demonstrated, and they have been reported to act combinatorially and synergistically to regulate grapevine STS genes (Vannozzi et al. 2018). For instance, *VviWRKY03* itself does not activate STS29, but in combination with *VviMYB14* significantly increases STS promoter activity. In addition, the APETALA2/ERF (AP2/ERF) and a bHLH TF are involved in the regulation of stilbene synthesis (Wong and Matus 2017; Vannozzi et al. 2018). Here we found structural differences between the *MYB14* promoters of Cabernet Sauvignon and Concord that may explain differences in the accumulation of stilbenes following Al^3+^ and UV-C treatments. We hypothesize that this may reflect the fact that the *MYB14* promoter from Concord has more regulatory *cis*-elements (Fig. 3) and TFBSs (Table S3) than the equivalent region from Cabernet Sauvignon. Moreover, the protective *cis-*element, ARE, which plays a role in the ROS signalling pathway, is absent from the promoter of *pVvMYB14* (Rushmore et al. 1991; Nguyen et al. 2009). Accordingly, this may explain why we observed earlier increases in the levels of ROS in Concord, which may in turn have contributed to the earlier activation of *MYB14*. Similarly, AT-Hook, WRKY, C2H2, Dof, GATA and MADF/Trihelix TFBSs (Boccacci et al. 2017) were identified in the promoter of *pVlMYB14*, which we propose may interact with *MYB14* to co-regulate the STS genes.

A specific group of NADP(H) oxidases located in the plasma membrane contribute to ROS release in plants (for review sees Marino et al. 2012) and NADP(H) oxidase can be activated by different stress conditions. ROS play an important role in the early signalling transduction in response to biotic and abiotic stresses (Neill et al. 2002). Our data support a model in which ROS act as an early signal to activate downstream genes, such *MYB14*, thereby promoting stilbene biosynthesis. In addition, the production of ROS is more pronounced and rapid in response to UV-C irradiation than following Al^3+^ treatment (Fig. 5 and Fig. S4). We hypothesize that Al^3+^ and UV-C may act through a similar defense pathway to activate stilbene synthesis in Concord, and that ROS may represent a common link, whereas the difference between types of treatment may reflect the timing of the ROS signal.

**Fig. 8:**
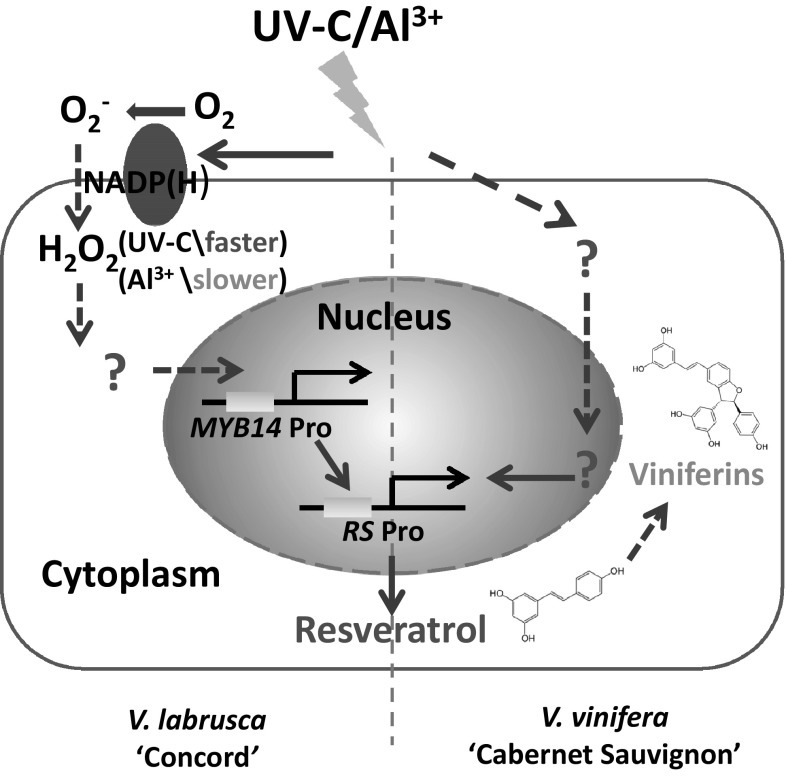
Model of defence responses triggered by Al^3+^ and UV-C in grapevine. Both treatments induce the accumulation of stilbenes, such as resveratrol and viniferins, to enhance the defence response in Concord by recruiting the activation of the transcription factor *MYB14*, but not in Cabernet Sauvignon. ROS plays an important role in the early signalling transduction to activate *pVlMYB14*. The production of ROS is induced more rapidly by UV-C than by the Al^3+^ treatment

We propose a model (Fig. 8) where ROS function as an early signal to induce *pVlMYB14* and consequently stilbene synthesis in Concord. The early synthesis of ROS triggered by Al^3+^ is relatively slower than that induced by the UV-C treatment. The mechanism by which the ROS signal activates *pVlMYB14* has not yet been elucidated. Duan et al. (2016) demonstrated that flg22 can activate the ROS, MAPK cascade and jasmonate signalling, which then converge on the *V. sylvestris MYB14* promoter in the cultivar Hoe29. In addition, it has been reported that the ROS, superoxide, enters the cell through aquaporins and causes actin bundling and detachment from the membrane, thereby triggering defence signalling and the activation of phytoalexin and stilbene synthesis genes (Chang et al. 2015; Eggenberger et al. 2016). The regulatory pathway involving ROS and *MYB14* is clearly complex and may involve other molecular signals (Zhang et al. 2007; Duan et al. 2016; Qiao et al. 2010; Chang et al. 2015; Eggenberger et al. 2016).

### Author contribution statement

DD conceived and designed the work. RB, YY-L, JL and KR-W performed the experiments. RB and DD analysed the data. DD wrote the manuscript. LX-W and GF-Z revised the manuscript. All authors gave final approval of the paper.

## Electronic supplementary material

Below is the link to the electronic supplementary material.

Supplementary material 1 (DOCX 47 KB)

Supplementary material 2 (DOCX 37 KB)

Supplementary material 3 (DOCX 46 KB)

Supplementary material 4 (DOCX 49 KB)

Supplementary material 5 (DOCX 21 KB)

Supplementary material 6 (DOCX 20 KB)

Supplementary material 7 (XLSX 15 KB)
