# Supplementary figures and images for: A specific allele of MYB14 in grapevine correlates with high stilbene inducibility triggered by Al3+ and UV-C radiation
Source: Plant Cell Rep. 2018 Oct 9;38(1):37–49. doi: 10.1007/s00299-018-2347-9 (PMC6320375; doi:10.1007/s00299-018-2347-9)

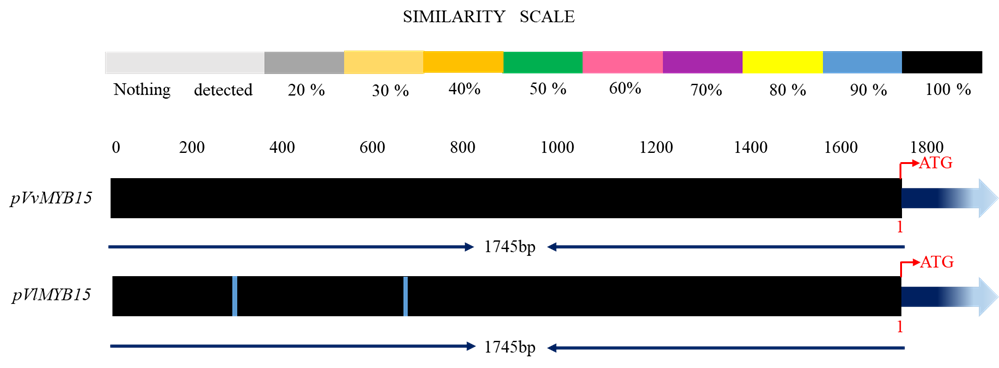


**Fig. S1** Similarities between the *MYB15* promoters from Concord and Cabernet Sauvignon.

Supplement: Supplementary file 1 — Supplementary material 1 (DOCX 47 KB) [file 299_2018_2347_MOESM1_ESM.docx]
