# Supplementary material for: A specific allele of MYB14 in grapevine correlates with high stilbene inducibility triggered by Al3+ and UV-C radiation
Source: Plant Cell Rep. 2018 Oct 9;38(1):37–49. doi: 10.1007/s00299-018-2347-9 (PMC6320375; doi:10.1007/s00299-018-2347-9)
Supplement: Supplementary file 2 — Supplementary material 2 (DOCX 37 KB) [file 299_2018_2347_MOESM2_ESM.docx]

**Cabernet sauvignon CTACTGACGTGC--------ACTAGCCTCTTTCTTTGACCCCTTCACACCATCGATGCTA 52**

**Concord CTACTGACGTGCACTAGCCTACTAGCCTCTTTCTTTGACCCCTTCACACCATCGATGCTA 60**

************** ******************************************

**Cabernet sauvignon AATTCCAAGCAGCCCAAATACTTCAACAAATGTGAACTGCACGTGTACACTCTCACACTC 112**

**Concord AATTCCAAGCAGCCCAAATACTTCAACAAATGTGAACTGCACGTGTACACTCTCACACTC 120**

****************************************************************

**Cabernet sauvignon GTGTCCATTTTGTGAATATGGTATTAGGTGT—TGTGTTAGGGTCTAATTTTGGGTCGAG 170**

**Concord GTGTCCATTTTGTGAATATGGTATTAGGTGTTGTGTGTTAGGGTCTAATTTTGGGTCGGG 180**

********************************* ************************* ***

**Cabernet sauvignon TTAAGAGAACACTTATTCATCATACTTTAGCTGGATATGGAAAGTTTTTGAATATGCAAT 230**

**Concord TTAAGAGAACACTTATTCATCATACTCAA----------AAAAGTTTTTGAAAATGTAAT 230**

***GT1-motif***

**************************** * ************ *** *****

**Cabernet sauvignon GAAGAAAAGGAAAGAAATTTATTTCAAATTTCATCCATTAGTATTTTTA----------- 279**

**Concord GAAGAAAAGGAAAGAAATTTATTTCAAATTTCATGGATTAGTACCTTTAAAAATTTATTT 290**

************************************ ******* ******

**Cabernet sauvignon ------------------------------------------------------------ 279**

**Concord CGAATGGTGTTTATTTTTTTGGTTTTTTGCTTAAAATAATTTATTTTCAGAAATTAGGTT 350**

***ARE* *MRE***

**Cabernet sauvignon ------------------------------------------------------------ 279**

**Concord ATTTGTTTTTCTACTTTTTTATGGCTTATTATAAATTTTTACTAAATAAAAAAAATCAAA 410**

**Cabernet sauvignon ------------------------------------------------------------ 279**

**Concord ATATGTAACTTTTTTTAAATAAAAAAATAACATATTGATTTTTTTTTTTACTTTTTAATA 470**

**Cabernet sauvignon ------------------------------------------------------------ 279**

**Concord CTTAATAGAAATAAAATACTACAAAAATAAACAACCTAATATTTAACACTATTAAACATT 530**

***AT-rich element MRE***

**Cabernet sauvignon -------------------------------------------------TAAATTTATTT 290**

**Concord AATATTCTATTTAGAATTAAGTTAAAAAACAAACACCACCTTCATTTATTAAATTTATTT 590**

***BOX 4*  *************

**Cabernet sauvignon TTTT-AAAGAGATTTAAATGAAAATATTTTAAGTAGAAGTGTTTAAAAATCCTCTAATAA 349**

**Concord TTTTAAAAGAGATTTAAATGAAAATAGTTTAAGTAGAAGTGTTTAAAAATCCTTTAATAA 650**

***F-box***

****** ********************* ************************** ********

**Cabernet sauvignon TTAAATTTTATTCTTTAAAAATTAAAATTTATATATATAAAATATATTTATCGGATGTAA 409**

**Concord TTAAATTTTATTCTTTAAAAATTAAAATTTATATATGTAAAATATATTTATAGGATGTAA 710**

************************************** ************** **********

**Cabernet sauvignon CCGGTAAAATTTGAAATATGTGACGATTTTATATATTTACATTAGGGTGGTGTTTATTTT 469**

**Concord CTGATAAAATTTGAAATATGTGACTACTTTATATATTTACACTAAGGTGATGTTTGTTTT 770**

***Box III***

*** * ******************** * ************** ** **** ***** ******

**Cabernet sauvignon TTGATTGAATAGAAAAAATCAAAATATTTGATTTTTCTAATTTAACTAAAAATAACTTGT 529**

**Concord TTGACTGAATAGAAAAAATTAAAATATTTGATTTTTTT--------TAAAAATAGTTTAT 822**

****** ************** **************** * ******** ** ***

**Cabernet sauvignon TCACATCGTTCAATATAACTAAACTGAATTTATTATTAATAGATTCAATTTAGTTATATT 589**

**Concord TGACATCATCCAATATAATTAAAATGAATTTGTTATTAATAGGTTCGATTTAATTATGTT 882**

*** ***** * ******** **** ******* ********** *** ***** **** ****

**Cabernet sauvignon GAAGAATATAAACGGATTACTTTTAACTGAATATAA—AAAATTAAGTATTTTAATTTTT 647**

**Concord GAAGGATGTGAATATGTTACTTTTAACTAAATAAAAATAAAATAAAATATTTTAATTTTT 942**

****** ** * ** ************ **** ** ***** ** ***************

**Cabernet sauvignon CATATTCAATAAAAAAACAAACATCACTT-------AAGATTCATATTATCAATGAAAAA 700**

**Concord -ATATTTAGTAAAAAAACAAACACCACCTAAACCTAAAGATCAAGGTTATCAATGAAAAA 1001**

***MRE circadian***

******* * ************** *** * ***** * ****************

**Cabernet sauvignon CTCAAAATTAAAATATTTCTGCATATATTCTAAGGCACCGCCCTAAGATGAGCCTGTTAT 760**

**Concord CTCAAAATTAAAATCTTTCTGCATATATTTTAAAGCACCGCCTTAAGATGAGCCCGTTAT 1061**

**************** ************** *** ******** *********** *******

**Cabernet sauvignon TCTATTAAAGAGATAATAACGAATTTGAAAAGGCAGAAAAGGAAAATACCAAGAAGGATT 820**

**Concord TCTATTAAAGAGATAATAACGAATTTGAAAAGGCAGAAAAGGAAAATACCAAGAAGGACT 1121**

************************************************************ ***

**Cabernet sauvignon TGGGGATATTGAACGTCACATTCATAGGGATCACCTTGCAGAAGGAAACAAAAACAAGAT 880**

**Concord TGGGGATATTGAACGTCACATTCATAGGGATCACCTTGCAGAAGAAAACAAAAATAAGAT 1181**

********************************************** ********* *******

**Cabernet sauvignon GAAATTCATTCCGGGTAAGTTATTATATAGCAAGTTGGTGCGTTAATTTGCCAAAGTTGG 940**

**Concord GAAATTCATTCCGCGTAAGTTATTATATGGCAAGTTGGTGCGTTAATTTGCCAAAGTTGG 1241**

*************** ************** *********************************

**Cabernet sauvignon TCACAAGTTTCATTAAAATAATAATAATAATAATAGGAAAGAAAGAAGGAAAAGAAAAAT 1000**

**Concord TCACAAGTTTCATTAAAATAATAATAATAATAATAGGAAAGAAAGAAGGAAAAGAAAAAT 1301**

****************************************************************

**Cabernet sauvignon TCTTGAACTCAAATGTAAAATATCTGAACATGCCCCAATTAATGGCCATGCTAGTTCAAG 1060**

**Concord TCTTGAACTCAAATGTAAAATATCTGAACATGCCCCAATTAATGGCCATGGTAGTTCAAG 1361**

**************************************************** ***********

**Cabernet sauvignon GAAAAGAAAAACCCCACGTTTTATTTGACCAATAAACAAACACTCGTGTCATCCAGTGAG 1120**

**Concord GAAAAGAAAAACCCCACGTTTTATTTGACCAATAAACAAACACTCGTGTCATCCAGTGAG 1421**

****************************************************************

**Cabernet sauvignon GCGGTTCCTAGCAATTGTGGGCTAAAAAGGATATGCCTTTTTATTTTCTTTTTTTTCTAA 1180**

**Concord GCGGTTCCTAGCAATTGTGGGCTAAAAAGGATATGCCTTTTTTTCTTC-TTTTTTTCTAA 1480**

***5UTR Py-rich stretch***

******************************************** * *** *************

**Cabernet sauvignon ATTCTATCAACAGTTGTTTGTACTATCTGCAAATTGCAGCAGCTGGACTCCTCATTATAA 1240**

**Concord ATTCTATCAACAGTTGTTTGTCCTATCTGCAAATTGCAGCAGCTGGACTCCTCATTATAA 1540**

***GATA-motif***

*********************** ****************************************

**Cabernet sauvignon ATACCCGCTCATGGGCTTCAAATCGGTTTGAGCTTGGGACATCAAGAGAGAACACAGAGA 1300**

**Concord ATACCCGCTCATGGGCTTCAAATCGGTTTGAGCTTGGGACATCGAGAGAGAACA------ 1594**

********************************************* ************

**Cabernet sauvignon TAAACAAGGGTGTCTCAGTCTCAAATCCTTACATAGAAAAGAAAAA 1346**

**Concord ---------------CAGTCTCAGATCCTTACATAGAGAAGAAAAA 1625**

********** ************* **********

**Fig. S2** Sequence alignment and differences in *cis*-elements between the *MYB14* promoters of Concord and Cabernet Sauvignon.

*TATA-box*: core promoter element around -30 of transcription start

*5UTR Py-rich stretch*: cis-acting element conferring high transcription levels

*Box 4*: part of a conserved DNA module involved in light responsiveness

*circadian*: cis-acting regulatory element involved in circadian control

*ARE*: cis-acting regulatory element essential for the anaerobic induction

*AT-rich element*: binding site of AT-rich DNA binding protein (ATBP-1)

*Box III*: protein binding site

*GATA-motif*: part of a light responsive element

*GT1-motif*: light responsive element

*MRE*: MYB binding site involved in light responsiveness
