# Supplementary material for: A specific allele of MYB14 in grapevine correlates with high stilbene inducibility triggered by Al3+ and UV-C radiation
Source: Plant Cell Rep. 2018 Oct 9;38(1):37–49. doi: 10.1007/s00299-018-2347-9 (PMC6320375; doi:10.1007/s00299-018-2347-9)
Supplement: Supplementary file 3 — Supplementary material 3 (DOCX 46 KB) [file 299_2018_2347_MOESM3_ESM.docx]

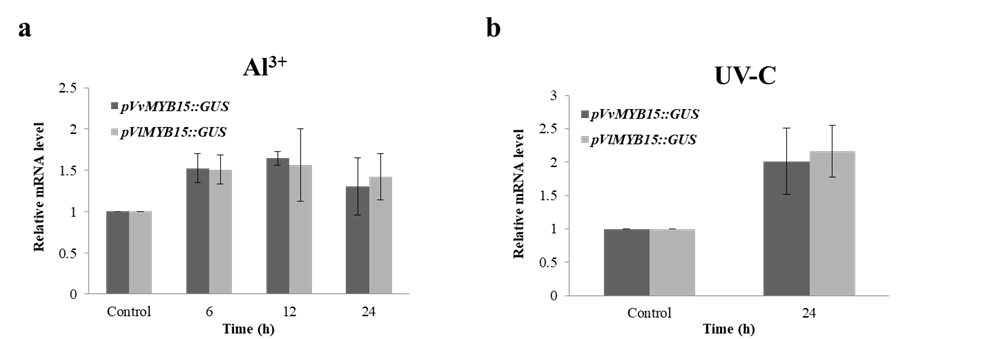


**Fig. S3** GUS transcripts levels (*pVlMYB15*::GUS and *pVvMYB15*::GUS) in response to a 1% Al^3+^ treatment at different time points (**a**) and at 24 h (**b**) after UV-C irradiation for 10 min, measured by RT-qPCR. Values indicate with mean values and standard errors from three independent experimental series.
