# Supplementary material for: A specific allele of MYB14 in grapevine correlates with high stilbene inducibility triggered by Al3+ and UV-C radiation
Source: Plant Cell Rep. 2018 Oct 9;38(1):37–49. doi: 10.1007/s00299-018-2347-9 (PMC6320375; doi:10.1007/s00299-018-2347-9)
Supplement: Supplementary file 4 — Supplementary material 4 (DOCX 49 KB) [file 299_2018_2347_MOESM4_ESM.docx]

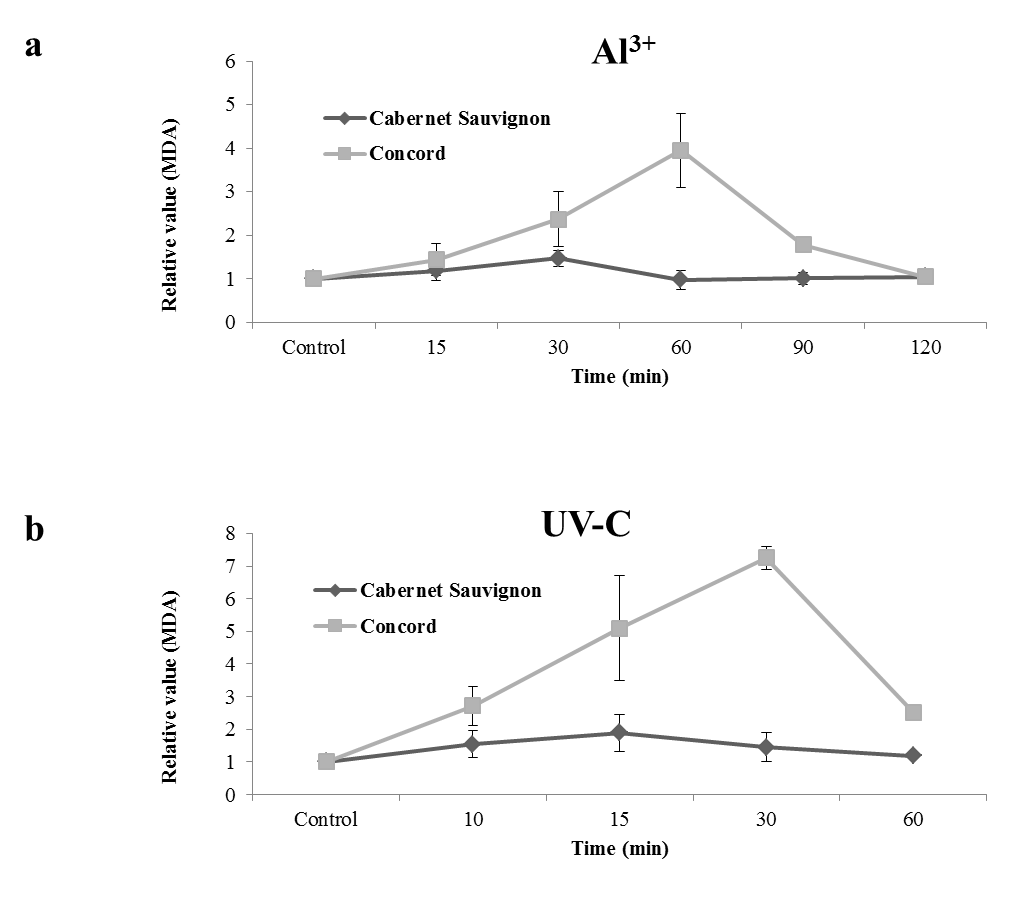


**Fig. S4** Relative amounts of malonedialdehyde (MDA) induced by 1% Al^3+^ (**a**) and 10 min UV-C (**b**) treatments in Cabernet Sauvignon and Concord. Leaves were incubated with streile water or not exposed to UV-C at the same time points as negative controls. Values represent means and standard errors from nine independent biological replicates.
