# Supplementary material for: A specific allele of MYB14 in grapevine correlates with high stilbene inducibility triggered by Al3+ and UV-C radiation
Source: Plant Cell Rep. 2018 Oct 9;38(1):37–49. doi: 10.1007/s00299-018-2347-9 (PMC6320375; doi:10.1007/s00299-018-2347-9)
Supplement: Supplementary file 5 — Supplementary material 5 (DOCX 21 KB) [file 299_2018_2347_MOESM5_ESM.docx]

**Table S1.** PCR primers used for the amplification of full length of *MYB14* and *MYB15* sequences in two genotypes.

| MYB14-F | 5’- CTACTGACGTGCACTAGCCT - 3’ |
| --- | --- |
| MYB14-R | 5’ -GCAGAGTGAAAGTGCAACACG -3’ |
| MYB15-F | 5’-GCCAAGGACTTGACTTGGAA-3’ |
| MYB15-R | 5’ -CTTCGATGACCAAATCTTTGAA -3’ |
| Vv-pMYB14-*HindⅢ-*F | 5’-*aagctt*TACTGACGTGCACTAGCCT-3’ |
| Vv-pMYB14*-BglⅡ*-R | 5’-GA*agatct*TTTTTCTTTTCTATGTAAGGATTTGAGACT-3’ |
| Vv-pMYB15-*HindⅢ-*F | 5’-*aagctt*GCCAAGGACTTGACTTGGAA-3’ |
| Vv-pMYB15-*BglⅡ-*R | 5’*-*GA*agatct*TCCTTGTTTGTTCTCCAATAGG-3’ |
| Vl-pMYB14-*HindⅢ-*F | 5’-*aagctt*CTACTGACGTGCACTAGCCT -3’ |
| Vl-pMYB14-*BglⅡ-*R | 5’-GA*agatct*TCTTTTTCTTCTCTATGTAAGGATCTGAGACT-3’ |
| Vl-pMYB15-*HindⅢ-*F | 5’-*aagctt*GCCAAGGACTTGACTTGGAA-3’ |
| Vl-pMYB15-*BglⅡ-*R | 5’ -GA*agatct* CTTGTTTGTTCTCCAATAGGAACA-3’ |
