# Supplementary material for: A specific allele of MYB14 in grapevine correlates with high stilbene inducibility triggered by Al3+ and UV-C radiation
Source: Plant Cell Rep. 2018 Oct 9;38(1):37–49. doi: 10.1007/s00299-018-2347-9 (PMC6320375; doi:10.1007/s00299-018-2347-9)
Supplement: Supplementary file 6 — Supplementary material 6 (DOCX 20 KB) [file 299_2018_2347_MOESM6_ESM.docx]

**Table S2.** Primers used for real-time quantitative PCR in this study.

| **Name** | **Primer sequence 5'-3'** | **Reference** |
| --- | --- | --- |
| *NbEF1-α* | Sense: 5’- -3’ AGAGGCCCTCAGACAAAC  Antisense: 5’- -3’ TAGGTCCAAAGGTCACAA | Zhang *et al*. (2015) |
| *GUS* | Sense: 5’- -3’ ATTATGCGGGCAACGTCTGGTATCAG  Antisense: 5’- -3’ CATCGGCTTCAAATGGCGTATAGC | Xu et al. (2010) |
| *EF1-α* | Sense: 5’- -3’ TGTCATGTTGTGTCGTGTCCT  Antisense: 5’- -3’ CCAAAATATCCGGAGTAAAAGA | Duan et al. (2015) |
| *PAL* | Sense: 5’- -3’ TGCTGACTGGTGAAAAGGTG  Antisense: 5’- -3’ CGTTCCAAGCACTGAGACAA | Belhadj et al. (2008) |
| *RS* | Sense: 5’- -3’ TGGAAGCAACTAGGCATGTG  Antisense: 5’- -3’ GTGGCTTTTTCCCCCTTTAG | Duan et al. (2015) |
| *MYB14* | Sense: 5’- -3’ GGGGTTGAAGAAAGGTCCAT  Antisense: 5’- -3’ GGCCTCAGATAATTCGTCCA | Duan et al. (2016) |
| *MYB15* | Sense: 5’- -3’ CAAGAATGAACAGATGGAGGAG  Antisense: 5’- -3’ TCTGCGACTGCTGGGAAA | Höll et al. (2013) |
